# Effects of Flooding-induced Changes in Bradyrhizobia Occupancy on the Growth of Adzuki Bean (*Vigna angularis*)

**DOI:** 10.1264/jsme2.ME25041

**Published:** 2025-11-13

**Authors:** Sokichi Shiro, Shundai Takei

**Affiliations:** 1 Institute of Agricultural and Life Sciences, Academic Assembly, Shimane University, 1060 Nishikawatsu-cho Matsue, Shimane 690–8504, Japan; 2 Department of Agricultural and Forest Sciences, Faculty of Life and Environmental Sciences, Shimane University, 1060 Nishikawatsu-cho Matsue, Shimane 690–8504, Japan

**Keywords:** adzuki bean, flooding, occupancy, growth, compatibility

## Abstract

We herein exami­ned the effects of soil flooding treatments on the occupancy of adzuki bean-nodulated bradyrhizobia and how changes in its occupancy affects adzuki bean growth. Microcosms containing mixtures of four different strains were prepared and incubated under flooded and non-flooded conditions. These microcosms were then used to cultivate adzuki bean in culture pots. After assessing the growth parameters of the plants, nodules collected from the roots were analyzed to assess occupancy rates. Be31, which exhibited a similar restriction fragment length polymorphism (RFLP) pattern in the 16S–23S rRNA gene ITS region to *Bradyrhizobium elkanii* USDA 31, was dominant overall. However, its occupancy declined under flooded conditions, while the occupancy of Bd110, similar to *Bradyrhizobium diazoefficiens* USDA 110^T^, increased. Furthermore, a non–metric multidimensional scaling anal­ysis showed that adzuki bean-nodulated bradyrhizobial communities were affected by changes in Bd110 and Be31 occupancies due to soil flooding. In terms of growth, shoot length and shoot dry weight generally increased in cultivars where Bd110 occupancy surpassed that of Be31 under flooding. A correlation anal­ysis revealed that Bd110 occupancy correlated with shoot dry weight. These results suggest that increased Bd110 occupancy through soil flooding enhanced adzuki bean growth. However, alternative methods need to be considered in order to more effectively regulate Be31 occupancy.

Adzuki bean (*Vigna angularis*) is predominantly produced in East Asian countries, including Japan, Korea, and China, as well as in non-East Asian countries, such as Canada, primarily for export to Japan. Hokkaido, located at Japan’s northern tip, is the main production region, accounting for more than 80% of the total cultivated area and more than 90% of the total harvest in Japan. The utilization and amino acid profile of adzuki bean are similar to‍ ‍those of kidney bean (*Phaseolus vulgaris* L.) (Duke, 1981; Robinson, 1983; Kimura *et al.*, 2004). In Europe and the United States, adzuki bean is recognized for its higher protein content, lower fat, natural sugar content, higher nutritional value, and better digestibility than kidney bean (Delić *et al.*, 2010). Moreover, adzuki bean contains proanthocyanidins, a class of polyphenols with superior radical scavenging activity to vitamin C, vitamin E, and β-carotene (Sato *et al.*, 2005). These attributes contribute to the status of adzuki bean as a globally demanded legume crop.

As a legume, adzuki bean engages in symbiotic nitrogen fixation with rhizobia, including members of the genera *Bradyrhizobium*, *Rhizobium*, and *Sinorhizobium* (Fujihara *et al.*, 2006; Shiro *et al.*, 2023). Adzuki bean has high nitrogen requirements and makes significant use of nitrogen obtained through fixation (Kimura *et al.*, 2004). Therefore, associations with rhizobia strains possessing superior nitrogen-fixing abilities are essential for enhancing adzuki bean productivity. Delić *et al.* (2010) conducted inoculation tests in cultivation pots using *Bradyrhizobium* strains for adzuki beans isolated from Serbian soil. Their findings showed that two *Bradyrhizobium japonicum* isolates significantly increased shoot nitrogen content over the non-inoculated control, indicating high nitrogen-fixing potential. Shiro *et al.* (2025) also performed field inoculation tests on adzuki bean with three *Bradyrhizobium diazoefficiens* strains isolated from Japanese soil to evaluate inoculation effects and competition with indigenous strains. No significant improvements were observed in growth or yield due to the inoculation, with indigenous *Bradyrhizobium elkanii* strains remaining dominant. Therefore, to enhance adzuki bean growth and yield through inoculations, it is necessary to increase the occupancy of *B. diazoefficiens* strains in order to outcompete native *B. elkanii*.

The *B. diazoefficiens* USDA 110^T^ and 122 strains are known to perform denitrification, the reduction of nitrate ions to nitrogen gas, under reducing soil conditions (Sameshima-Saito *et al.*, 2006; Shiina *et al.*, 2014; Saeki *et al.*, 2017). The genes *napAB*, *nirK*, *norCB*, and *nosZ* are involved in the denitrification process (Bedmar *et al.*, 2005; Sameshima-Saito *et al.*, 2006; Siqueira *et al.*, 2014) and are harbored by *B. diazoefficiens* USDA 110^T^ (Sameshima-Saito *et al.*, 2006; Shiina *et al.*, 2014). This strain, which carries the *nosZ* gene, has been reported to predominate in alluvial and flooded soils (Shiina *et al.*, 2014; Saeki *et al.*, 2017). The *B. diazoefficiens* isolates used in the study by Shiro *et al.* (2025) possessed the *nosZ* gene and exhibited similar restriction fragment length polymorphism (RFLP) patterns in the 16S–23S rRNA gene internal transcribed spacer (ITS) region to *B. diazoefficiens* USDA 110^T^. Therefore, the inoculation of soil with these strains, followed by flooding and adzuki bean cultivation, may suppress infection by *B. elkanii*, which lacks a full denitrification capacity, and increase the occupancy of inoculated strains with complete denitrification functionality. *B. diazoefficiens* strains possessing the *nosZ* gene have been reported to result in a higher chlo­rophyll content in leaves and a higher shoot nitrogen content in soybean than *B. japonicum* and *B. elkanii*, which lack this gene (Brignoli *et al.*, 2024). Based on these findings, we hypothesized that soil flooding may increase the occupancy of *nosZ*-carrying strains, thereby enhancing adzuki bean growth and yield.

Therefore, the present study investigated whether *nosZ*-carrying strains, associated with complete denitrification, achieve greater occupancy under flooded conditions than other strains. To test this hypothesis, microcosms combining multiple *Bradyrhizobium* strains were established. Additionally, plant growth assessments were performed to examine the effects of changes in nodulating strain occupancy on the growth of adzuki bean.

## Materials and Methods

### Preparation of microcosm soil

To examine the effects of soil flooding on the occupancy of adzuki bean-nodulated bradyrhizobia and adzuki bean growth, we conducted a cultivation test using microcosms containing selected indigenous isolates. Four isolates, AN1, AN9, AN18, and AN22, were used as test strains. These strains were originally isolated from the root nodules of adzuki bean plants cultivated in soil collected from Izumo Shimane Prefecture, Japan (35°25′50.5″ N, 132°50′00.2″ E). A RFLP anal­ysis of the 16S–23S rRNA gene ITS region revealed that AN1 showed a similar RFLP pattern to *B. elkanii* USDA 31, while AN9 resembled *B. diazoefficiens* USDA 110^T^ and carried the *nosZ* gene. AN18 and AN22 exhibited similar RFLP patterns to *B. japonicum* USDA 115 and 123, respectively. AN1 and AN9 were used in a previous adzuki bean study (Shiro *et al.*, 2025). These isolates were cultured in Yeast–Mannitol liquid medium (K_2_HPO_4_ 0.5 g, MgSO_4_·7H_2_O 0.2 g, NaCl 0.1 g, Yeast Extract 0.4 g, and D-(–)-Mannitol 10 g, adjusted to 1 L with distilled water, pH 6.8, autoclaved at 121°C for 20‍ ‍min) (Vincent, 1970) at 28°C for 7 days with shaking. The cultures were then mixed with sterile commercial soil (Sun·Soil s NS-3; Nagata) (primarily andosol; pH [H_2_O]: 5.27, EC: 0.12‍ ‍mS‍ ‍m^–1^, NO_3_^–^–N: 14.2‍ ‍mg kg^–1^, and NH_4_^+^–N: 30.4‍ ‍mg kg^–1^), also autoclaved at 121°C for 20‍ ‍min, to achieve a bacterial density of approximately 10^6^ cells [g‍ ‍dry‍ ‍soil]^–1^. The microcosms containing the mixed strains were incubated overnight and then subjected to either flooded or non-flooded treatments. Under flooded conditions, the soil was saturated with sterile distilled water, maintaining a free water level 2‍ ‍cm above the soil surface. In non-flooded conditions, soil moisture was maintained at 40% of the maximum water-holding capacity. Microcosms were incubated at 25°C for 4 weeks, with the periodic addition of sterile distilled water to maintain respective water levels. In the subsequent cultivation test, two additional treatments were included: sterile soil without any isolates (negative control, N–control) and microcosms where isolates were mixed with sterile soil and incubated overnight (positive control, P–control). In N–control and P–control, the flooding and non-flooding treatments were not performed, and microcosms that were simply mixed with sterile distilled water and a bacterial solution with isolates, respectively, were used.

### Measurement of the oxidation–reduction potential (Eh)

The Eh values of the flooded and non-flooded microcosms were measured throughout the incubation period. A portable soil pH/nitrate/Eh meter (PRN–41; FUJIWARA SCIENTIFIC) was used for these measurements. Platinum electrodes were inserted into the designated microcosms, and Eh values were recorded periodically on days 3, 6, 10, 14, and 28 after the start of incubation.

### Cultivation test of adzuki bean

Adzuki bean was cultivated in 1/5000-a Wagener pots filled with a mixture of vermiculite and culture soil (primarily andosol) at a ratio of 60 L to 20 kg, respectively. Three cultivars were used: Iwate-dainagon, Hokuto-dainagon, and Tanba-dainagon. Vermiculite and culture soil (Sun·Soil s NS-3; Nagata) were commercially available products sterilized during manufacturing. A chemical fertilizer containing 3% N, 10% P, and 10% K was applied at 2‍ ‍g per pot. Adzuki bean seeds were sown on August 24, 2021. Prior to sowing, seeds were sterilized by soaking in 70% ethanol for 30‍ ‍s and then in a 2.5% sodium hypochlorite solution (0.25% available chlorine) for 3‍ ‍min, followed by rinsing with sterile distilled water. Approximately 2–3‍ ‍g of microcosm material was placed into the culture soil at a depth of 2–3‍ ‍cm, and the seeds were sown directly above it. The pot experiment followed a two-factor factorial design (cultivar×treatment) with four replicates per treatment. Each pot contained two plants and was placed in an outdoor environment. Adzuki bean seeds were cultivated for 8 weeks with irrigation applied as needed. After 8 weeks, the following growth parameters were recorded: shoot length, number of nodes, shoot dry weight, and number of nodules. Shoots were dried at 80°C for 72‍ ‍h using a drying oven. Nodules collected from the roots were stored at –‍20°C for further anal­yses.

### Analysis of occupancy of adzuki bean-nodulated bradyrhizobia

To estimate the effects of soil flooding on the occupancy of adzuki bean-nodulating bradyrhizobia, total DNA was extracted from root nodules, and occupancy was assessed by a PCR–RFLP anal­ysis of the 16S–23S rRNA gene ITS region. DNA for PCR templates was directly extracted from individual nodules, following a previously described method (Shiro *et al.*, 2016), based on the protocol by Hiraishi *et al.* (1995). Each nodule was homogenized in 50‍ ‍μL of BL buffer (40‍ ‍mM Tris-HCl, 1% Tween 20, 0.5% Nonidet P-40, and 1‍ ‍mM EDTA, pH 8.0), 40‍ ‍μL of sterile distilled water, and 10‍ ‍μL of proteinase K (1‍ ‍mg mL^–1^) and the mixture was then incubated using a dry bath incubator at 60°C for 20‍ ‍min and at 95°C for 5‍ ‍min. After centrifugation at 15,000‍ ‍rpm for 10‍ ‍min, the supernatant was collected in a new microtube and used for PCR amplification of the 16S–23S rRNA gene ITS region. PCR was conducted using Tks Gflex^TM^ DNA Polymerase (Takara Bio). The primer set used for amplification was BraITS-F (5′-GACTGGGGTGAAGTCGTAAC-3′) and BraITS-R (5′-ACGTCCTTCATCGCCTC-3′) (Saeki *et al.*, 2004). The PCR cycle included an initial denaturation at 94°C for 3‍ ‍min, followed by 30 cycles at 98°C for 10‍ ‍s, 60°C for 15‍ ‍s, and 68°C for 30‍ ‍s, with a final extension at 68°C for 2‍ ‍min. In the RFLP anal­ysis, the amplified PCR product was digested with *Msp*I (Takara Bio) at 37°C for 16 h. DNA fragments were separated by electrophoresis on a 3% agarose gel and visualized using Midori Green Xtra (FastGene^®^, NIPPON Genetics). To identify the RFLP patterns of adzuki bean-nodulated bradyrhizobia, the four reference isolates used in the microcosm mixtures, namely, AN1, AN9, AN18, and AN22, were employed.

### Non-metric multidimensional scaling (NMDS) anal­ysis and statistical anal­ysis

To examine the effects of soil flooding on changes in the adzuki bean-nodulated bradyrhizobial community, we performed a NMDS anal­ysis based on the Bray–Curtis dissimilarity (Shiro *et al.*, 2012; Saeki *et al.*, 2017). The NMDS anal­ysis was conducted by R version 4.2.2 (R Core Team, 2022) using the ‘metaMDS’ function including ‘vegan’ package version 2.6-4 (Minakata *et al.*, 2023).

Statistical anal­yses were performed using R version 4.2.2 (R Core Team, 2022). To evaluate the effects of the flooding treatment and cultivar on the growth of adzuki beans and the occupancy of adzuki bean-nodulated bradyrhizobia, a two-way ANOVA followed by multiple comparison tests was conducted using anovakun version 4.8.9 (Iseki, 2023), which operates within R. Additionally, correlation coefficients between growth parameters and the occupancy of adzuki bean-nodulated bradyrhizobia were calculated, and the significance of the relationships was tested using ‘psych’ package version 2.3.3 in R.

## Results

### Changes in Eh and temperature information

**Fig. 1.**
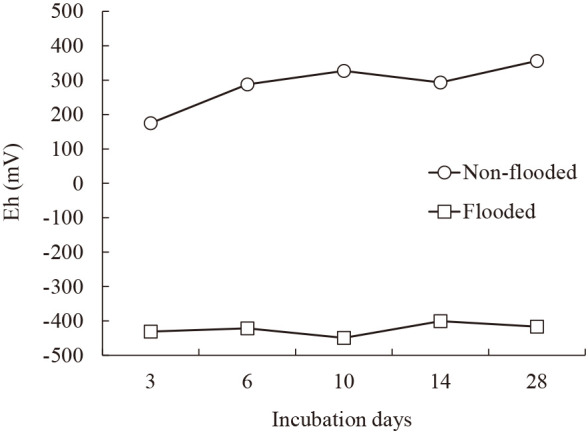
Changes in the oxidation–reduction potential of microcosms under flooded and non-flooded conditions.

Changes in Eh during the incubation period are shown in Fig. 1. In non-flooded microcosms, Eh values were 175.0‍ ‍mV at 3 days, 288.0 mV at 6 days, 327.0 mV at 10 days, 293.3 mV at 14 days, and 356.0 mV at 28 days. In contrast, flooded microcosms recorded Eh values of –‍431‍ ‍mv at 3 days, –421.3‍ ‍mV at 6 days, –449.3 mV at 10 days, –400.7 mV at 14 days, and –416.7 mV at 28 days. These measurements confirmed oxidative conditions in non-flooded microcosms and reducing conditions in flooded microcosms.

Temperature data during the cultivation period are shown in Table 1. Average monthly temperatures were 26.5°C in August, 23.6°C in September, and 18.1°C in October. Temperatures in September and October exceeded the annual average. Maximum and minimum daily average temperatures for each month showed similar changes.

### Effects of the flooding treatment on occupancy and the community structure

The occupancy of nodulated bradyrhizobia in adzuki bean cultivated in microcosms under various treatments is shown in Table 2. Be31, which shows a similar RFLP pattern to AN1, was dominant, followed by Bd110, which shows a similar RFLP pattern to AN9, and Bj115, which shows a similar RFLP pattern to AN18. Bj123, which shows a similar RFLP pattern to AN22, was not detected in the present study. The N-control treatment involved microcosms without introduced isolates but infected with bradyrhizobia derived from tap water or sand dust. An anal­ysis of variance showed no significant differences by cultivar; however, significant differences by treatment were observed for Be31, Bd110, and Bj115. No interaction effects were detected. Although Be31 occupancy exceeded 60% in the N-control, P-control, and non-flooded treatments, it was generally lower under flooded conditions than under non-flooded conditions. Bd110 occupancy was 27.3% in P-control and 27.2% under non-flooded conditions, but increased to 38.9% under flooded conditions. Hokuto-dainagon and Tanba-dainagon generally had higher Bd110 occupancy than Be31 under flooded conditions. Bj115 occupancy did not significantly differ between flooded and non-flooded conditions.

**Fig. 2.**
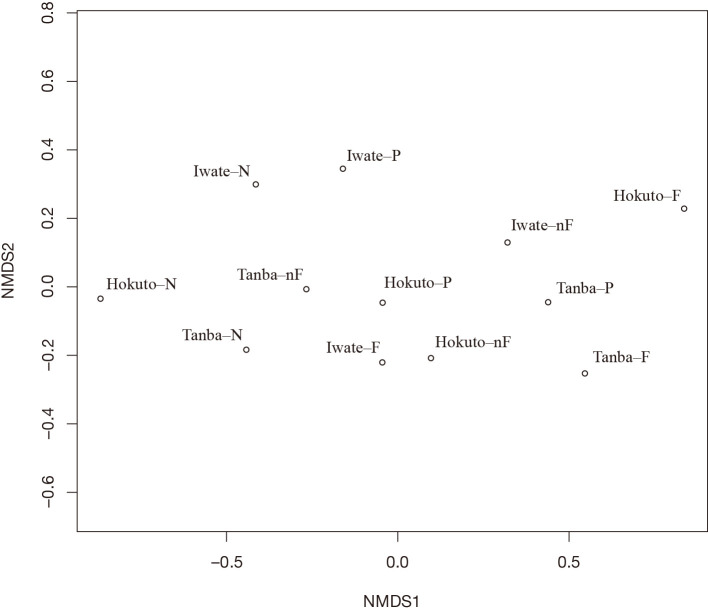
Non–metric multidimensional scaling (NMDS) plot based on the Bray–Curtis dissimilarity of adzuki bean-nodulated bradyrhizobial communities. Iwate–, Hokuto–, and Tanba– indicate the cultivars Iwate-dainagon, Hokuto-dainagon, and Tanba-dainagon, respectively, while N, P, nF, and F indicate the treatments N-control, P-control, non-flooded, and flooded, respectively.

The NMDS plot of adzuki bean-nodulated bradyrhizobial communities is shown in Fig. 2. In each cultivar, changes in the community were detected due to the flooding and non-flooding treatments. Communities with a high Be31 occupancy shifted to the left of the center, while those with increased Bd110 occupancy shifted to the right of the center.

### Effects of changes in occupancy on the growth of adzuki bean

The growth parameters of adzuki beans cultivated in microcosms with various treatments are shown in Table 3. An anal­ysis of variance revealed significant differences by cultivar for shoot length, node number, and nodule number, and by treatment for node number and shoot dry weight. No interaction effects were detected. Among the cultivars, Iwate-dainagon showed significantly shorter shoot length, and Hokuto-dainagon had significantly fewer nodes and nodules than the other cultivars. Regarding treatments, N-control resulted in significantly fewer nodes than the non-flooded treatment and lower shoot dry weight than P-control. Shoot dry weight was generally higher under flooded conditions in Hokuto-dainagon and under both P-control and flooded conditions in Tanba-dainagon than the other treatments.

The results of the correlation anal­ysis between the occupancy data of Be31 and Bd110 and growth parameters are presented in Table 4. Scatter plots of pairs showing correlations are shown in Fig. S1. The occupancy of Be31 negatively correlated with all parameters and showed negative correlations with Bd110 occupancy (–0.956, *P*<0.001) and shoot dry weight (–0.582, *P*<0.05). Conversely, Bd110 occupancy showed positive correlations with growth parameters, including a positive correlation with shoot dry weight (0.726, *P*<0.01). Additionally, positive correlations were found between shoot dry weight and node number (0.596, *P*<0.05), between nodule number and node number (0.838, *P*<0.001), and between nodule number and shoot dry weight (0.721, *P*<0.01).

## Discussion

In the present study, microcosms were used to investigate whether high nitrogen-fixing strains with a complete denitrification capacity compete with other strains and increase their occupancy under flooded soil conditions. Although the microcosm environment differs from that in the field, we also exami­ned the behavior of indigenous strains and their compatibility with various adzuki bean cultivars by using different indigenous strains and multiple cultivars.

In the anal­ysis of adzuki bean-nodulated bradyrhizobia occupancy, the flooding treatment generally increased the occupancy of Bd110 (Table 2). A flooding treatment has been suggested to increase nodule occupancy by promoting the dominance of Bd110, such as AN9, which possesses the *nosZ* gene, in soil under anaerobic conditions (Saeki *et al.*, 2017; Siqueira *et al.*, 2017). Furthermore, the NMDS anal­ysis showed that communities changed with increases and decreases in Bd110 and Be31 occupancies under flooding and non-flooding conditions (Fig. 2). However, Be31 remained dominant across most treatments, with the exception of the flooded treatments for Hokuto-dainagon and Tanba-dainagon, where the occupancy rates of Bd110 and Be31 were reversed (Table 2). These results indicate that soil flooding promotes the occupancy of bradyrhizobia strains with a complete denitrification capacity, such as AN9, and affects the adzuki bean-nodulated bradyrhizobial community. However, we also found that in some cultivars, such as Iwate-dainagon, the occupancy of Bd110 did not increase or surpass that of Be31, even under flooding conditions. One possible reason for this is compatibility with the bradyrhizobial strain using for the inoculation. Soybean, which forms symbiotic relationships with *Bradyrhizobium* species, such as adzuki bean (Christopher *et al.*, 2018; Ayalew and Yoseph, 2020; Pereira *et al.*, 2020; Gough *et al.*, 2021; de Oliveira *et al.*, 2022; Shiro *et al.*, 2023), possesses the *Rj* gene, which restricts symbiosis with some *Bradyrhizobium* strains (Hayashi *et al.*, 2012). This gene has also been reported to affect the community structure of soybean-nodulated bradyrhizobia (Minami *et al.*, 2009; Shiro *et al.*, 2012). Although it remains unclear whether adzuki bean carries a gene analogous to *Rj*, previous studies on *Bradyrhizobium* strains incompatible with legumes of the genus *Vigna* (Nguyen *et al.*, 2017; Nguyen *et al.*, 2020) suggested that adzuki bean exhibits varying degrees of compatibility with different bradyrhizobia. Therefore, it is necessary to select strains similar to *B. diazoefficiens* that possess the *nosZ* gene, such as AN9, which are more compatible with various adzuki bean cultivars and more competitive than strains similar to *B. elkanii* USDA 31, such as AN1. In a test cultivating adzuki bean seeds in pots with treated microcosms, significant differences were observed in shoot length, node number, and nodule number between cultivars, while significant differences between treatments were noted for node number and shoot dry weight (Table 3). Additionally, in the occupancy anal­ysis, Bd110 was dominant under flooded conditions in Hokuto-dainagon, as well as under flooded conditions in Tanba-dainagon (Table 2), and these treatments increased shoot length and shoot dry weight (Table 3). Furthermore, the correlation anal­ysis between occupancy and growth parameters revealed that Bd110 occupancy positively correlated with all parameters (Table 4) and showed a positive correlation with shoot dry weight (Fig. S1C). A positive correlation was also detected between nodule number and shoot dry weight (Table 4 and Fig. S1F). These results suggest that increased Bd110 occupancy enhanced adzuki bean growth and that achieving a sufficient nodule number was also important for growth improvement. In a field cultivation study on adzuki beans, Shiro *et al.* (2025) reported positive correlations between nodule number and both shoot dry weight and yield as well as between shoot dry weight and yield. Furthermore, adzuki beans were found to depend more on nitrogen obtained through symbiotic nitrogen fixation than on nitrogen in the soil (Kimura *et al.*, 2004), and a positive correlation was reported between shoot nitrogen content and shoot dry weight (Delić *et al.*, 2010; Shiro *et al.*, 2025). Although this study did not examine the relationship between shoot nitrogen content and shoot dry weight, the increase in shoot dry weight observed in this study is presumed to result from an increased shoot nitrogen content due to greater Bd110 occupancy, indicating that maintaining the occupancy of high nitrogen-fixing strains and achieving adequate nodule formation are necessary for improving the growth and yield of adzuki beans.

The present results suggest that flooding the soil prior to adzuki bean cultivation enhanced plant growth by increasing the occupancy of high nitrogen-fixing strains with a complete denitrification capacity in the soil, such as AN9. However, this approach did not fully suppress the occupancy of competing strains, such as AN1. In the present study, nitrate, which is used as a substrate for denitrification, was not added to the microcosms. In contrast, Saeki *et al.* (2017) reported the addition of potassium nitrate to microcosms in their experiments. The addition of nitrate, along with an incubation for four weeks or longer, may further increase the occupancy of strains such as AN9 in the soil, potentially enhancing their presence during adzuki bean cultivation. This aspect warrants further investigation to develop the method into a more practical cultivation technique.

## Citation

Shiro, S., and Takei, S. (2025) Effects of Flooding-induced Changes in Bradyrhizobia Occupancy on the Growth of Adzuki Bean (*Vigna angularis*). *Microbes Environ ***40**: ME25041.

https://doi.org/10.1264/jsme2.ME25041

## Supplementary Material

Supplementary Material

## Figures and Tables

**Table 1.** Temperature data during the cultivation period.

Month	Average (°C)		
	Temperature	Max. daily temperature	Min. daily temperature
Aug.	26.5 (27.1)	30.6 (31.6)	23.6 (23.8)
Sept.	23.6 (22.9)	27.4 (27.1)	20.8 (19.6)
Oct.	18.1 (17.4)	23.1 (22.0)	14.2 (13.4)

Temperature data were obtained from the Japan Meteorological Agency website. Values in parentheses represent the average for the period 1991–2020.

**Table 2.** Effects of the flooding treatment on the occupancy (%) of adzuki bean-nodulated bradyrhizobia.

Cultivar	Treatment	Be31	Bd110	Bj115	Bj123
Iwate-dainagon	N-control	98.2±1.8	1.8±1.8	0.0±0.0	0.0±0.0
	P-control	85.4±6.9	13.0±7.2	1.6±1.6	0.0±0.0
	Non-flooded	58.2±10.3	32.8±12.9	9.0±5.9	0.0±0.0
	Flooded	54.8±8.3	26.2±6.8	19.0±5.2	0.0±0.0
Hokuto-dainagon	N-control	100.0±0.0	0.0±0.0	0.0±0.0	0.0±0.0
	P-control	73.1±9.1	24.5±8.2	2.4±1.4	0.0±0.0
	Non-flooded	54.6±11.4	29.3±16.7	16.1±6.5	0.0±0.0
	Flooded	40.3±7.5	48.0±10.7	11.7±4.2	0.0±0.0
Tanba-dainagon	N-control	81.2±6.3	9.4±6.0	9.4±6.0	0.0±0.0
	P-control	52.4±13.4	44.5±13.6	3.1±3.1	0.0±0.0
	Non-flooded	68.6±12.9	19.4±9.1	12.0±4.2	0.0±0.0
	Flooded	38.0±11.4	42.4±17.3	19.6±9.6	0.0±0.0
Iwate-dainagon		74.2±5.8	18.4±4.8	7.4±2.7	0.0±0.0
Hokuto-dainagon		67.0±6.8	25.5±6.5	7.5±2.4	0.0±0.0
Tanba-dainagon		60.1±6.6	28.9±6.7	11.0±3.2	0.0±0.0
	N-control	93.2±3.2 a	3.7±2.2 b	3.1±2.2 b	0.0±0.0
	P-control	70.3±6.7 b	27.3±6.6 a	2.4±1.1 b	0.0±0.0
	Non-flooded	60.5±6.3 bc	27.2±7.1 a	12.3±3.1 ab	0.0±0.0
	Flooded	44.3±5.3 c	38.9±7.0 a	16.8±3.7 a	0.0±0.0
ANOVA	Cultivar	ns	ns	ns	ns
	Treatment	***	**	**	ns
	C×T	ns	ns	ns	ns

Isolates showing similar restriction fragment length polymorphism (RFLP) patterns to AN1, AN9, AN18, and AN22 were designated as Be31, Bd110, Bj115, and Bj123, respectively. Values represent the mean±standard error (*n*=4). Asterisks indicate significant differences at *P<0.05, **P<0.01, and ***P<0.001. Significant differences between groups marked with different letters were exami­ned using the Holm–Bonferroni method (*P*<0.05).

**Table 3.** Effects of flooding-induced changes in bradyrhizobial occupancy on adzuki bean growth.

Cultivar	Treatment	Shoot Length (cm plant^–1^)	Node number (No. plant^–1^)	Shoot dry weight (g plant^–1^)	Nodule number (No. plant^–1^)
Iwate-dainagon	N-control	13.2±0.8	3.9±0.4	1.5±0.1	37.3±16.5
	P-control	15.2±0.9	4.3±0.5	2.1±0.3	62.8±12.5
	Non-flooded	15.1±0.6	5.3±0.1	2.1±0.2	73.4±20.4
	Flooded	13.2±0.1	4.1±0.3	1.6±0.3	44.1±7.8
Hokuto-dainagon	N-control	19.5±0.4	3.1±0.2	1.7±0.2	24.3±9.0
	P-control	19.9±0.9	3.3±0.1	1.9±0.3	26.3±4.8
	Non-flooded	19.6±0.3	3.8±0.3	1.7±0.2	23.6±4.8
	Flooded	20.5±0.5	4.3±0.3	2.1±0.1	35.5±5.7
Tanba-dainagon	N-control	16.1±0.7	4.1±0.1	1.7±0.1	35.6±4.8
	P-control	18.2±0.4	4.6±0.2	2.4±0.1	67.3±3.7
	Non-flooded	16.7±0.7	4.3±0.3	1.9±0.1	60.1±6.5
	Flooded	17.7±0.7	4.6±0.3	2.3±0.3	81.0±10.9
Iwate-dainagon		14.2±0.4 c	4.4±0.2 a	1.8±0.1	54.4±7.7 a
Hokuto-dainagon		19.8±0.3 a	3.6±0.2 b	1.9±0.1	27.4±3.1 b
Tanba-dainagon		17.2±0.4 b	4.4±0.1 a	2.1±0.1	61.0±5.3 a
	N-control	16.3±0.9	3.7±0.2 b	1.6±0.1 b	32.4±6.1
	P-control	17.8±0.7	4.0±0.3 ab	2.1±0.1 a	52.1±6.9
	Non-flooded	17.1±0.6	4.4±0.2 a	1.9±0.1 ab	52.4±9.2
	Flooded	17.1±0.9	4.3±0.2 ab	2.0±0.2 ab	53.5±7.4
ANOVA	Cultivar	***	***	ns	***
	Treatment	ns	*	*	ns
	C×T	ns	ns	ns	ns

Values represent the mean±standard error (*n*=4). Asterisks indicate significant differences at *P<0.05, **P<0.01, and ***P<0.001. Significant differences between groups marked with different letters were exami­ned using the Holm–Bonferroni method (*P*<0.05).

**Table 4.** Correlation coefficients between the occupancy of adzuki bean-nodulated bradyrhizobia and growth parameters.

	Be31	Bd110	SL	NoN	SDW	NdN
Be31	1.000					
Bd110	–0.956***	1.000				
SL	–0.281	0.374	1.000			
NoN	–0.545	0.555	–0.358	1.000		
SDW	–0.582*	0.726**	0.329	0.596*	1.000	
NdN	–0.410	0.429	–0.340	0.838***	0.721**	1.000

Be31, Bd110, SL, NoN, SDW, and NdN represent the occupancy of Be31, the occupancy of Bd110, shoot length, node number, shoot dry weight, and nodule number, respectively. Asterisks indicate correlations at * *P*<0.05, ** *P*<0.01, and *** *P*<0.001, respectively.
