# Supplementary material for: Effects of Flooding-induced Changes in Bradyrhizobia Occupancy on the Growth of Adzuki Bean (Vigna angularis)
Source: Microbes Environ. 2025 Nov 13;40(4):ME25041. doi: 10.1264/jsme2.ME25041 (PMC12727202; doi:10.1264/jsme2.ME25041)
Supplement: Supplementary file 1 — Supplementary Material [file 40_25041_s1.pdf]

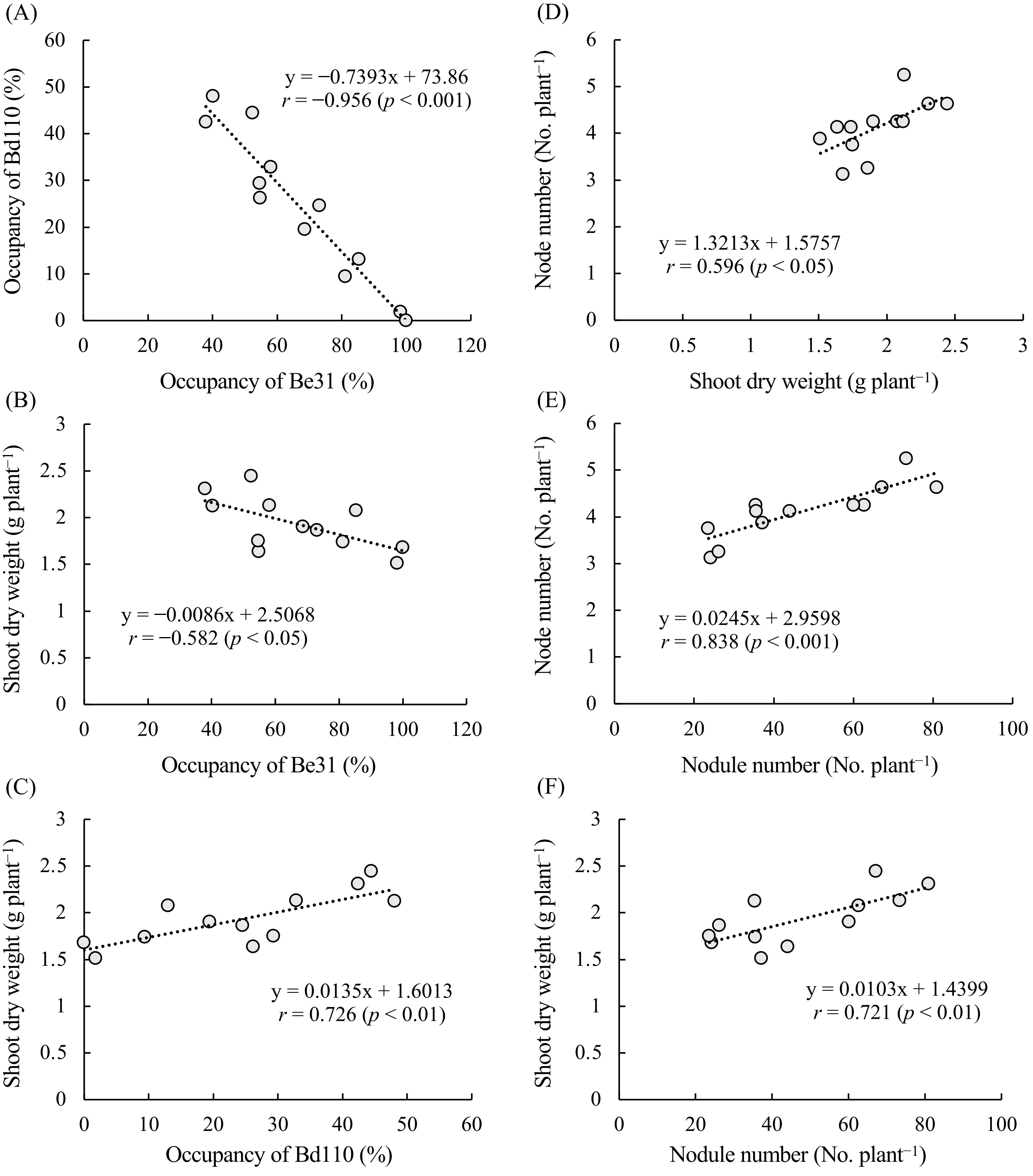

Fig. S1. Correlation coefficients between occupancy of Be31 and occupancy of Bd110 (A), occupancy of Be31 and shoot dry weight (B), occupancy of Bd110 and shoot dry weight (C), shoot dry weight and node number (D), nodule number and node number (E), and nodule number and shoot dry weight (F).
